# Society Meetings

**Published:** 1912-05

**Authors:** 


### SOCIETY MEETINGS

---

### Medical Society of the County of Erie.

The regular meeting of this Society was held Monday, April 22, 1912, in the Buffalo Library Building.

President Thomas H. McKee called the meeting to order at 8.30 p. m. Secretary F. C. Gram read the minutes of the regular meeting held February 19, 1912, and the minutes of the Council meetings held March 4th, March 9th and April 1, 1912, all of which were approved.

Dr. C. A. Wall, Chairman of the Committee on Membership recommended the re-instatement of Dr. Cora B. Lattin, who had resigned on removing from Erie County. He also recommended the election to membership of the following: Drs. Francis J. Lennon, Michael A. Conboy, Natalie K. Mankell, Frederick E. Sperry and James I. Kearney.

The secretary was directed to cast the vote of the society for each, and they were thereupon declared duly elected.

Dr. Wm. H. Thornton, Chairman of the Special Committee appointed for the purpose of devising means for the better collection of delinquent accounts, outlined the work thus far and, on his motion, the committee was authorized to expend a sum not to exceed twenty dollars ($20.00) in promoting its work.

Dr. Thornton also reported for the delegates to the recent meeting of the State Society.

Dr. F. S. Crego called the attention of the Society to the State Law which prohibits the keeping of insane patients at Police Stations.

He, therefore, moved that the President appoint a committee of five for the purpose of conferring with the Health Commissioner and other proper authorities, with a view to estabalishing a suitable psychopathic ward in this city.

The motion was carried and the President appointed Drs. Crego, Matzinger, Sharp, Putnam and Nairn as such committee.

President McKee called attention to the system of cleaning street cars, by which conductors, on some lines, are compelled to sweep their cars at the end of the line instead of having them cleaned in the car barns. This creates a nuisance which the passengers are compelled to suffer. On motion of Dr. Lytle, this matter was referred to the committee on Public Health.

Dr. Hartwig presented a patient, a Polish laborer about 40 years old, on whom violent spells of vomiting could be incited by pressure on a certain part on the side of his neck.

Following was the scientific program:

Malposition of the Uterus during the Puerperium.
Dr. F. C. Goldsborough.

Differential Diagnosis of Haematuria.
Dr. David C. Wheeler.

Preliminary Report on Conservation in the Treatment of Prostatic Hypertrophy.
Dr. Nelson W. Wilson.

Treatment of Compound Fractures.
Dr. Thew Wright.

Each paper was discussed, after which adjournment followed.

---

The 106th Annual Meeting of the Medical Society of the State of New York, was held in Albany, Tuesday, April 16, to Thursday, April 18, 1912. As detailed reports will be published in the Society's Journal, only some general impressions of the meeting will be given. For the first time, the work was divided among sections, 1. Medicine, 2. Surgery, 3. Eye, Ear, Nose and Throat, 4. Mental and Nervous, including Eugenics and Expert Testimony, 5. Public Health and Preventive Medicine. In a sense, the division of the society is to be regreted but, with a registration of about 550, and with almost overy man actually in attendance, the old idea of a single body is no longer practicable. Another innovation was the institution of entertainments for ladies, and the reception, dance and supper, at the Ten Eyck on Wednesday. With the exhibits of drugs, *x*-ray coils buzzing, and the free dispensing of grape juice, the general effect was of an A. M. A. meeting on a small scale.

Semi-popular lectures in the evening, in the Assembly Chamber drew regretably small audiences. The medical men, fatigued by long technical programs and inadequate ventilation were, for the most part inclined to rest and visit, rather than to attend lectures which would afford comparatively little new information, while, for some reason or other, the public did not respond enthusiastically to the invitation.

Joint sessions of sections, were held on Vertigo, Poliomyelitis, and Hyperthyroidea. Short symposiums were provided in the several sessions in addition to unrelated papers. In five halfday sessions, from 28 to 39 papers were scheduled for each section, often with pre-arranged discussions. Comparatively few readers failed to appear and still fewer completed either paper or discussion in the time limit set. Most of the papers were full of meat, and several were illustrated with diagrams, stereopticon, etc. We can't refrain from urging that, at the next meeting, a desperate effort be made to have all rooms fairly well ventilated and, that, at the risk of hurting some one's feelings, much smaller programs be provided, to allow full discussion, and to prevent the encroachment of other, perhaps necessary and always desirable, private conferences, on the regular programs.

Except in the tendency to follow the precedent of the A. M. A., the change of the State Society from a permanent senate of the profession, to an ipso facto, general membership, is scarcely noticeable. To one who has attended many such meetings, there was conspicuous, as elsewhere, the gradual change in the personnel and appearance of the assemblage. At the same time, Albany was entertaining men who manufacture, sell, and use, big excavating and building machinery. A generation ago, even if one failed in an individual case, he could instantly have distinguished a little group of business men from one of physicians. Now, one had to look close and then be in doubt. The pompous professional dress and carriage have gone, the man who was prominent in the profession, not from technical knowledge but from self-assertiveness and local success, has vanished, the youth who puffed with the rudiments of a fresh science, disdained the practical physician, has grown up and his successors are more open minded. There were undoubtedly many country doctors at the meeting but the only man who would have passed as a type, we were assured lived in Manhattan. Altogether, it was an earnest, hard working, modest, but self-respecting lot of men conforming more and more closely to a common external type, but with plenty of individuality inside of their skulls.

---

THE Buffalo Civic League favored the citizens of that city with a stereopticon lecture on Smoke Abatement, April 16. Mr. J. M. Searle, Chief Smoke Inspecor of Pittsburg, was the lecturer. Dr. Lucien Howe presided.

---

THE Buffalo Medical and Surgical League held its monthly meeting at the Hofbräu, April 11. Dr. Francis M. O'Gorman read a paper on brain tumors, exhibiting one weighing about 90 grams, successfully removed. Lunch was served.

---

THE Western New York Homeopathic Medical Society recently held a purely social meeting at the Hotel Statler, in Buffalo, with vaudeville and a banquet, following a session of hard scientific work. Officers were elected as follows: Dr. H. W. Hoyt of Rochester, President; Dr. George R. Critchlow of Buffalo, Dr. B. D. Shed of Arcade, Vice-Presidents; Dr. R. Montfort Schley of Buffalo, Secretary-Treasurer.

---

THE American Gastro-enterological Association will hold its 16th annual meeting at Atlantic City, June 3 and 4.

---

THE Michigan Health Officers' Association will meet at Ann Arbor, May 22 and 23.

---

THE American Proctologic Society will hold its 14th annual meeting at Atlantic City, at the Hotel Chalfonte, June 3 and 4. Only one member is listed in the special territory of this journal: Dr. Dwight H. Murray of Syracuse, who will read a paper on Pruritus Ani.

---

THE American Academy of Medicine will meet at Atlantic City, Friday, May 31 to Sunday, June 2. Suicide in the Public Press, Hygiene in the Public Schools, Immigration Problems, Relation of Women to Modern Industrialism, Delinquent Girls, are some of the topics. Physicians interested in medical sociology, education, etc., and eligible by virtue of college degrees, are invited to become members.

---

THE MONROE COUNTY SANITARY ASSOCIATION held its regular quarterly meeting on Tuesday, April 16, and listened to a paper by Dr. W. S. Magill, of the State Hygienic Laboratory, on the "Production of Milk." There was a very large attendance.

---

THE BLACKWELL MEDICAL SOCIETY of Rochester, a society of women physicians held its monthly meeting Friday, April 19, at Iola Sanitarium. The last meeting of the season is on June 13 and is the occasion for holding the annual picnic. Dr. Mary A. Nickerson is the president.

---

THE ROCHESTER ACADEMY OF MEDICINE has discontiued its weekly meetings. The regular meeting of the society will be held probably May 8, although the council has power to change the date. The four sections meet Wednesday evenings as their officers determine. Dr. C. E. Darrow is president, and Dr. E. L. Hanes is secretary. The Academy is about to remove its library to new quarters just leased on the ground floor of 355 East

Avenue, near Alexander Street. The meeting in the future will therefore be held in the permanent home of the Academy.

---

THE ROCHESTER ACADEMY OF SCIENCE holds its last meeting of the season Monday May 3. This is to be an "experience meeting" and for exhibition of collections.

---

THE MEDICAL SOCIETY OF THE COUNTY OF MONROE will hold its annual all-day meeting on Tuesday, May 21st. Dr. S. W. Little will preside and Dr. A. C. Snell is secretary. An interesting program is being prepared.

---

THE HOSPITAL MEDICAL SOCIETY of Rochester holds its final meeting of the year on Thursday evening, April 25.

---

THE ROCHESTER PATHOLOGICAL SOCIETY continues its fortnightly meetings on Thursdays as follows:

May 2—Dr. E. A. French.
May 16—Dr. E. A. Shumway.
May 30—Dr. Wesley Mulligan.
June 15—"Annual Excursion and Picnic."

